# Upcycling of poultry protein hydrolysates using membrane filtration technology – Effects on sensory properties and chemical composition

**DOI:** 10.1016/j.fochx.2026.104181

**Published:** 2026-07-08

**Authors:** Tone Aspevik, Elin Katinka Dankel, Shaun Leivers, Nils Kristian Afseth, Åge Oterhals, John-Erik Haugen, Marianne Skov, Mari Øvrum Gaarder

**Affiliations:** a Nofima, P.O. Box 1425 Oasen, N-5844, Bergen, Norway; b Nofima, P.O. Box 210, N-1431, Ås, Norway; c Norilia AS, P.O. Box 396 Økern, N-0513. Oslo, Norway

**Keywords:** Poultry protein hydrolysates, Membrane filtration, Microfiltration, Nanofiltration, Sensory properties, Volatile compounds

## Abstract

Processing poultry meat generates large amounts of protein-rich side streams. Enzymatic hydrolysis is widely used for upcycling but often produces bitter and unpalatable products. Membrane filtration may improve quality, but its effects on sensory properties and composition remain unclear. This study examined microfiltration (MF), nanofiltration (NF), and diafiltration of a poultry protein hydrolysate using sensory analysis, ^1^H NMR metabolomics, and volatile compounds. MF removed lipids and suspended particles, reducing flavour intensity and lipid oxidation products, with hexanal reduced by 94–96%. NF reduced ash and low-molecular-weight metabolites while increasing protein levels. Despite these changes, NF had limited additional sensory effects and slightly increased bitterness, likely related to reduced salt levels and increased peptide concentration. Altogether, these findings highlight the role of matrix effects in flavour perception and support strategies to improve the sensory quality of poultry protein hydrolysates.

## Highlights

• Microfiltration (MF) reduced flavour intensity by removing lipids and particles.
• Nanofiltration (NF) altered composition with minor additional sensory effects.
• Bitterness increased after NF, likely due to salt removal and peptide enrichment.
• Sensory changes reflect interactions within the hydrolysate matrix.
• Findings support improved processing of poultry side streams.

## Graphical abstract

**Figure:**
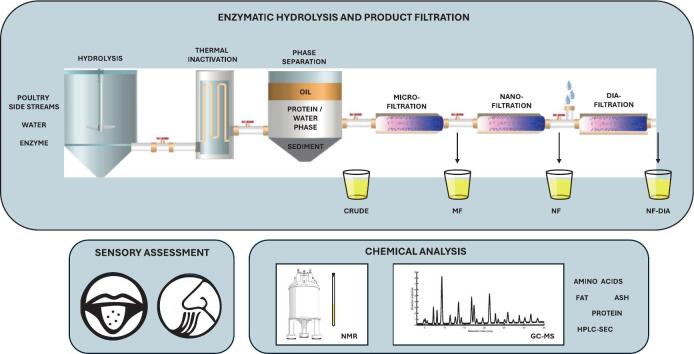

## 1 Introduction

The world poultry meat production increased from 9 to 133 million tonnes between 1961 and 2020 (FAOSTAT, 2025). Poultry processing results in considerable amounts of side streams, such as blood, bones, feathers, offal and trimmings (Seidavi et al., 2019). While a major proportion of these materials are currently converted into low-value feed ingredients, improved processing strategies may enable their upcycling into higher value food or nutraceutical ingredients.

Enzymatic protein hydrolysis is widely applied to transform poultry side streams into protein hydrolysates with favorable nutritional profiles and potential bioactive properties (Aspevik et al., 2017; Romero-Garay et al., 2022; Sorokina et al., 2023). However, a drawback with enzymatic protein hydrolysates is their strong and often unpalatable tastes and flavours, including bitterness and off-flavours, which restrict their application in products for human consumption. As taste and flavour are primary drivers of consumer interest in nutraceutical products (Cardello & Schutz, 2003), producing as taste-neutral products as possible is preferred.

The sensory properties of protein hydrolysates are influenced by multiple factors, including peptide composition, free amino acids, low-molecular weight metabolites, and lipid-derived compounds (Ramakrishna et al., 2026). Bitterness in protein hydrolysates is primarily driven by small, hydrophobic peptides released during hydrolysis, with both amino acid composition and sequence strongly influencing the perception threshold (Liu et al., 2022; Zhang & Liu, 2024). In contrast, other sensory attributes associated with protein hydrolysates are more strongly influenced by the intrinsic composition of the raw material and its storage conditions and can be more challenging to address. In poultry-based hydrolysates, volatile compounds and small metabolites originating from raw material composition, storage conditions, and processing history have been shown to contribute substantially to characteristic flavour profiles (de Souza Cunha et al., 2023; Steinsholm et al., 2020).

Poultry side streams are relatively rich in fats and lipids (Five et al., 2024), which can act as carriers of intrinsic flavour compounds and are susceptible to oxidation. Lipid oxidation products, particularly aldehydes such as hexanal, are well known contributors to off-flavours described as grassy, rancid, or vegetable-like (Han et al., 2023; Leksrisompong et al., 2010). Hexanal is widely used as an indicator of lipid oxidation and product quality (Li et al., 2024), and its presence has been linked to negative sensory perception in protein-rich foods. Consequently, processing strategies that effectively remove lipids and associated oxidation products may be critical for improving the sensory quality of poultry protein hydrolysates.

Membrane filtration techniques have emerged as an effective downstream technology to refine protein hydrolysates and modulate their chemical composition (Ghalamara et al., 2024; Saidi et al., 2014). Microfiltration (MF) is commonly used to refine protein hydrolysates by removal of suspended particles and fat, whereas nanofiltration (NF) can be used to remove small ions and organic molecules from the products. NF also removes water, reducing downstream processing costs associated with dewatering of the hydrolysate. Diafiltration may be applied in combination with MF or NF to further enhance purity, although at the expense of increased water and energy use (Steinsholm et al., 2021). Despite increasing interest in membrane filtration, most studies have focused on peptide fractionation and bioactivity, with limited emphasis on sensory properties, and even fewer studies have compared the relative sensory impact of MF versus NF. Further, the mechanistic links between filtration-induced compositional changes and sensory perception remain poorly understood, particularly for poultry protein hydrolysates.

To date, only Steinsholm et al. (2021) have systematically assessed sensory changes in membrane filtered fish protein hydrolysates, where NF was reported to reduce sensory intensity by removing small water-soluble compounds. Whether similar effects apply to poultry protein hydrolysates, remains unclear. The present study addresses this knowledge gap by investigating the effects of MF, NF and diafiltration on the chemical composition, metabolite profiles, volatile compounds, and sensory properties of a commercial poultry protein hydrolysate. By combining sensory analysis with ^1^H NMR metabolomics and volatile compound analysis, this study aims to elucidate key chemical drivers underlying sensory changes induced by membrane filtration. These findings may contribute to improved processing strategies and the development of more sensory-acceptable protein hydrolysates from poultry side streams.

## 2 Materials and methods

### 2.1 Materials

Enzymatic protein hydrolysate concentrate (60% dry matter), based on poultry side streams, was provided by Bioco, Hærland, Norway. The concentrate was stored cold (4 °C) upon processing. Peptide standards were purchased from Sigma Aldrich (Oslo, Norway) except lysozyme (Fluka biochemicals, Buchs, Switzerland) and Alberta standards (Alberta Peptide Institute, Department of Biochemistry, University of Alberta, Edmonton, Canada). All chemicals for analysis were of analytical grade.

### 2.2 Methods

#### 2.2.1 Chemical methods

Analysis of nitrogen (N) was performed according to the Kjeldahl method (*ISO* 5983-2, 2009) and crude protein was estimated by multiplying by a factor of 6.25. Ash was determined by sample combustion at 550 °C (*ISO* 5984-2, 2002). Fat content was determined by use of chloroform-methanol extraction (Bligh & Dyer, 1959). Dry matter was determined by drying at 103 °C (ISO 6496-2, 1999). Amino acid composition was determined according to AOAC Official Method 2018.06 (Jaudzems & Fuerer, 2022) with some adjustments. The samples were hydrolyzed in 6 M HCl for 22 h at 110 °C. L-Norvaline (Sigma-Aldrich) was added as internal standard. The UPLC analysis was conducted on a Waters ACQUITY UPLC (ultra performance liquid chromatography) I-Class PLUS with fluorescence detector (Waters, Milford, MA, USA). The separation of the amino acids was done on a Waters AccQ-Tag Ultra column (2.1 × 100 mm, with a particle size of 1.7 μm) (Waters, Milford, MA, USA). For derivatization of amino acids, 6-aminoquinolyl-N-hydroxysuccinimidyl carbamate (AQC) supplied by Waters (Waters AccQ-Tag Ultra derivatization kit, Waters, Milford, MA, USA) was used. Waters AccQ-Tag Ultra eluent A and B (Waters, Milford, MA, USA) were used as mobile phases. The flow rate was 0.7 ml/min and the solvent gradient was set as follows with curve 6 unless mentioned otherwise: 0.0–0.54 min 99.9% A and 0.1% B; 0.54–5.74 min 90.9% A and 9.1% B (curve 7); 5.74–7.74 min 78.8% A and 21.2% B; 7.74–8.04 min 40.4% A and 59.6% B; 8.04–8.05 min 10% A and 90% B; 8.05–8.64 min 10% A and 90% B; 8.64–9.00 min 99.9% A and 0.1% B; 9.00–10.00 min 99.9% A and 0.1% B. The column temperature was set to 43 °C to ensure optimal separation of all amino acids including Hydroxyproline. The detection of amino acids was carried out using a fluorescence detector with wavelengths for excitation and emission set at 266 and 473 nm, respectively. The total analysis time was 10 min. Cysteine and cystine were determined after performic acid oxidation. Estimation of molecular weight distribution was performed by size exclusion chromatography (1260 series HPLC Agilent Technologies, Santa Clara, CA) as described by Oterhals and Samuelsen (2015).

#### 2.2.2 Membrane filtration

The protein hydrolysate concentrate was diluted to around 200 l with a dry matter concentration of about 7% prior to MF. After dilution, the hydrolysate was immediately subjected to filtration without any intermediate storage. A 0.1 μm ceramic filter apparatus (MT Separation, Flekkefjord, Norway) was used and the temperature was kept at approximately 60 °C throughout the filtration process. The MF permeate was further refined by NF using a spiral membrane with MWCO of 200 Da (TNF 3838, Toray Industries, Poway, CA). The retentate was concentrated to half volume before the addition of 1.5 volume of tap water to assess the effect of diafiltration. The membrane filtration experiments were performed as exploratory production trials with limited material availability. As a result, key operating parameters (e.g., transmembrane pressure, flow rate, and permeate flux) were not systematically controlled or monitored, and the process was operated under practical conditions with adjustment of flux and recirculation of retentate to maximize product recovery rather than process optimisation.

#### 2.2.3 NMR

^1^H NMR analysis was conducted on the same solutions used in the sensory evaluation, i.e., 2% (*w*/w) hydrolysate in tap water. Three 2 ml aliquots of each sample were transferred to Eppendorf microcentrifuge tubes and heated at 50 °C for 30 min (ThermoMixer F1.5, Eppendorf, Hamburg, Germany) to ensure complete dissolution of the hydrolysate, particularly the gelatin component. The samples were centrifuged (Micro star 17R, VWR International, Radnor, PA, USA) at 13,000 rpm for 5 min at 40 °C to remove any solid particles. After centrifugation, the still-warm supernatants were filtered through a Millex-HV PVDF syringe filter (0.45 μm pore size, Merck Millipore, Burlington, MA, USA). A 400 μl aliquot of the filtrate was mixed with 200 μl of an NMR stock solution containing 0.3 M potassium phosphate buffer (pH 7.0), D_2_O, 0.05 wt% TMSP (20%), and 0.3 mg/ml sodium azide. 550 μl aliquots of the buffered samples were transferred into 5 mm NMR tubes (DURAN, DVK Life Sciences, Wertheim, Germany).

The NMR spectra were acquired using a Bruker Avance Neo 400 NMR spectrometer (Bruker BioSpin, Billerica, MA, USA) operating at a proton frequency of 400.13 MHz. The ^1^H NMR spectra were collected using the standard Bruker pulse sequence *noesygppr1d* with water signal suppression achieved through presaturation pulses during the relaxation delay. The spectra were automatically phased, baseline corrected, and referenced to TMSP for x-axis calibration, before being imported into MATLAB for further analysis using the *rbnmr* script (Nyberg, 2025). Peak assignments were aided by in-house libraries and the Chenomx NMR Suite (Chenomx, Edmonton, Canada).

#### 2.2.4 Volatile organic compounds

Volatile organic compounds were analyzed using an automated dynamic headspace purge and trap system with a thermal desorption unit (Gerstel DHS-TDU-MPS, Gerstel GmbH & Co. KG, Mühlheim, Germany) interfaced with a gas chromatograph (Agilent 6890, Palo Alto, CA, USA) coupled with a mass spectrometer (Agilent 2977B) (HS-GC/MS system). Samples (1 g; 2% (*w*/w) dry matter concentration) were transferred to a 20 ml sample vial, flushed with nitrogen, sealed with a screw cap with Teflon septum and transferred to the sample exchanger of the HS-GC/MS instrument. Ethyl heptanoate in methanol (> 99%, Sigma-Aldrich Chemie GmbH, Steinheim, Germany) was added as an internal standard (0.4 μg) to the sample vial. The sample vials were incubated (70 °C) with shaking for 5 mins. Volatile compounds were dynamically extracted and trapped on the adsorbent tube with active charcoal (Tenax GR, particle size 60/80 mesh, Alltech Associates Inc., Deerfield, IL, USA) for 2 min at 100 ml/min, total 200 mL gas volume. Then the adsorbent sample tubes were back-flushed with nitrogen (100 ml/min) for 20 min for the removal of water. The volatile compounds were transferred into the gas chromatograph by thermal desorption. The compounds were separated on a polar DB-WAXetr gas chromatographic column (J & W Scientific/Agilent, 0.25 mm i.d., 0.5 μm film, 30 m). Helium (99.9999%) was used as the carrier gas. The following temperature program was used: 30 °C (10 min.) – 1 °C/min. to 40 °C (0 min.) – 3 °C/min (0 min.) to 70 °C (0 min.) – 6.5 °C/min to 230 °C (5 min.). Mass spectra were recorded in positive scan mode (*m*/*z* = 33–500) after electron impact ionization at 70 eV. The integration of peak areas was performed with the Mass Hunter software (Version 10.0, Agilent Technologies). The identification of compounds was confirmed by comparison of the measured mass spectra of the GC peaks with pure standards according to the NIST14L mass spectral library (version 2.2, 2014, US Commerce/Agilent). Two replicates of each sample were analyzed, and the precision of replicates was within 15%.

#### 2.2.5 Sensory evaluation

The hydrolysates were diluted to 2% (*w*/w) dry matter concentration with tap water and assessed by a sensory panel of 10 assessors at Nofima (Ås, Norway). The panel consists of employees specifically recruited and trained for sensory analysis, and participation is part of their contractual role. All participants provided informed consent to take part in the sensory evaluation and for the use of the collected data. Formal ethical approval was not required for this type of study in accordance with applicable institutional and national guidelines. All procedures were conducted in compliance with the Norwegian Research Ethics Act and relevant international ethical principles. All personal data were anonymised, and all participant identifiers were replaced with coded information. The assessors are regularly trained and tested in accordance with *ISO* 8586, 2012, which includes continuous training with references of the basic tastes, in addition to other attribute references where applicable. The panel is also regularly provided with a wide range of hydrolysates to obtain consensus on hydrolysate sensory attributes. A generic descriptive analysis was performed as described by Lawless & Heymann, 2010 and in accordance with ISO 13299., 2016. The evaluated attributes, typical for the hydrolysate samples to be tested, and their descriptions are listed in Supplementary Table 1. The assessors were calibrated on samples that were considered the most different in a pretest session. Samples were served in plastic glasses (20 ml) with a lid at room temperature (20 ± 2 °C) under red light, coded with a three-digit number in a randomized and balanced design. The attributes were evaluated on an unstructured 15-cm line scale with labeled end points from no intensity (1) to high intensity (9) and registered in a computer system for direct recording of data (EyeQuestion, Software Logic8 BV, Utrecht, the Netherlands). Tap water and unsalted crackers were available for palate cleansing during the assessment. Each sample was evaluated in duplicate.

#### 2.2.6 Statistical analysis

Analysis of variance (ANOVA) was performed using Minitab (v19.2, Pennsylvania State University, PA, USA). For levels of small metabolites from NMR profiling, one-way ANOVA was used. For the sensory data, a two-way mixed effects ANOVA model was conducted to assess differences between products for all sensory attributes. The product was set as a fixed variable, whereas assessor and interaction effects were set as random variables. Tukey's pairwise comparison was applied where significant (*p* < 0.05) differences were found. Multivariate NMR data analysis was performed in MATLAB using the PLS_Toolbox 9.3 (2023) (Eigenvector Research, Manson, WA, USA). The Aspen Unscrambler multivariate statistics software (version 14.5, Aspen Technology Inc., Bedford, MA, USA) was used for the correlation analysis (PLS) of the organic volatile compounds and sensory data. Segmented cross validation was applied for the prediction modelling.

## 3 Results and discussion

### 3.1 Chemical composition of hydrolysates

The proximate composition of the crude hydrolysate and products after filtration were evaluated to assess the effect of MF and NF (Table 1). The crude hydrolysate contained 6% lipids on dry matter basis, which were removed in the MF process. Conversely, the protein concentration, on dry matter basis, was increased in the MF-permeate, and even further increased after NF and diafiltration (NF-dia), which demonstrate that the filtration processes mainly removed amounts of non‑nitrogen containing dry matter. Furthermore, the levels of ash were reduced after NF and reflected the desalting properties of the membrane used, as also observed for fish protein hydrolysates (Steinsholm et al., 2021). The peptide molecular weight distribution of the products (Table 1) revealed a slight shift in peptide size distribution after NF, where the fraction of the smallest peptides <200 Da was reduced. Conversely, the relative content of fraction of peptides >200 Da increased in the NF and diafiltrated (NF-dia) hydrolysates. The amino acid composition of the hydrolysate before filtration (crude; Table 2) revealed a balanced amino acid distribution, including all essential amino acids. After MF, only minor differences in the total amino acid content were observed, except for a very slight increase in tryptophan and decrease in hydroxyproline, proline and glycine. In contrast, NF increased the levels of all amino acids on a dry matter basis due to the removal of non-protein dry matter, with the exception of tryptophan. Based on the assumption that collagen consists of 13.5% hydroxyproline (Lindberg et al., 2021), the collagen-derived peptides in the crude sample was estimated at 37%, with a slight reduction to 33% after MF (Table 2). However, after NF and diafiltration, the proportion increased again to 37%, suggesting a selective removal of non-collagenous free amino acids during the filtration process.

**Table 1:** Chemical composition of poultry protein hydrolysates before filtration (crude), after microfiltration (MF) and nanofiltration (NF) with diafiltration (NF-dia), on dry matter basis (g/100 g).

	Crude	MF	NF	NF-dia
Protein (N × 6.25)	87.6	90.4	95.5	100.0
Ash	7.6	8.0	4.9	3.0
Lipids	6.0	<0.1	<0.1	<0.1

*Peptide size distribution (Da)*				
> 15,000	1.2	0.9	0.8	0.9
15,000–10,000	13.9	12.3	10.6	10.3
10,000–8000	7.7	7.2	6.3	6
8000–6000	8.4	7.9	7.8	7.5
6000–4000	7.7	7.5	8.4	8.8
4000–2000	11.4	11.4	12.5	13.1
2000–1000	10.8	11.2	12.2	12.8
1000–500	8.3	8.8	10	10.7
500–200	7.9	8.5	9.1	9.8
< 200	22.7	24.3	22.4	20.1

**Table 2:** Amino acid composition of poultry protein hydrolysates before filtration (crude), after microfiltration (MF) and nanofiltration (NF) with diafiltration (NF-dia), on dry matter basis (g/100 g).

	Crude	MF	NF	NF-dia
EAA^1^				
Arginine	5.8	5.9	7.1	7.2
Histidine	1.9	1.8	2.1	2.2
Isoleucine	2.7	2.7	3.1	3.2
Leucine	5.2	5.3	5.7	5.8
Lysine	6.1	6.3	7.1	7.8
Methionine	1.8	1.9	2.0	2.0
Phenylalanine	2.5	2.5	2.6	2.7
Threonine	3.0	3.2	3.4	3.6
Tryptophan	0.8	0.9	0.6	0.7
Valine	3.1	3.2	3.5	3.7
Sum EAA	*32.9*	*33.6*	*37.1*	*38.9*

NEAA^2^				
Alanine	6.4	6.4	7.1	7.2
Aspartic acid	6.9	7.0	7.7	8.4
Cysteine	0.7	0.7	0.7	0.7
Glutamic acid	13.5	13.3	14.7	15.6
Glycine	9.9	9.7	10.9	11.4
Hydroxyproline	4.3	3.8	4.6	5.0
Proline	6.1	6.0	7.1	7.2
Serine	3.3	3.3	3.5	3.6
Tyrosine	1.7	1.6	1.9	1.9
*Sum NEAA*	*52.6*	*51.9*	*58.0*	*60.9*
Sum AA	85.5	85.6	95.1	99.8
Collagen^3^	37%	33%	35%	37%

contains 13.5% Hydroxyproline (Lindberg et al., 2021).

1 Essential amino acids; ^2^Non-essential amino acids; ^3^Based on the assumption that collagen.

### 3.2 Effect of membrane filtration on sensory properties

To evaluate the effect of the MF and NF processes on the sensory properties of the poultry hydrolysates, a generic descriptive analysis was conducted. After filtration, a clear and transparent product was produced, in contrast to more turbid crude hydrolysate. The sensory assessment of the products (Table 3) revealed a notable reduction of smell, taste and flavour intensities after the MF step, whereas only minor additional effects were shown during the successive NF-process. This suggests that fats and lipids and suspended particles likely act as dominant carriers and amplifiers of flavour perception in the assessed hydrolysates.

**Table 3:** Sensory properties of poultry protein hydrolysates before filtration (crude), after microfiltration (MF) and nanofiltration (NF) with diafiltration (NF-dia), evaluated at 2% dry matter concentration. Different letters indicate statistical difference (p < 0.05) among the products by two-way ANOVA and Tukey's multiple comparison test.

	Crude	MF	NF	NF-dia	*p-value*
Total smell	6.1^a^	5.2^b^	5.1^b^	5.0^b^	<0.001
Vegetable smell	2.3^b^	3.8^a^	3.9^a^	3.7^a^	0.003
Meaty smell	6.2^a^	3.5^b^	3.6^b^	3.1^b^	<0.001
Process smell	1.9	1.8	2.4	1.7	0.184
Roasted smell	3.4^a^	2.6^b^	2.8^ab^	2.7^ab^	0.014
Rancid smell	3.1^a^	1.9^b^	1.9^b^	1.4^b^	<0.001
Total taste and flavour	5.6	5.2	5.6	5.5	0.435
Sweet taste	2.5	2.7	2.7	2.7	0.616
Salt taste	2.9	2.9	2.7	2.8	0.650
Sour taste	2.3^b^	2.6^ab^	2.6^ab^	2.7^a^	0.032
Bitter taste	4.4^b^	4.7^ab^	4.8^ab^	5.1^a^	0.048
Umami taste	3.7	3.6	3.8	3.8	0.724
Vegetabe flavour	2.6^b^	4.5^a^	4.0^a^	4.3^a^	0.002
Meaty flavour	5.8^a^	3.1^b^	3.4^b^	3.2^b^	<0.001
Process flavour	2.5	2.8	2.9	2.5	0.326
Roasted flavour	3.2^a^	2.4^ab^	2.2^b^	2.3^ab^	0.023
Rancid taste	3.2^a^	1.9^b^	1.9^b^	1.5^b^	<0.001
Fatness	3.7^a^	3.2^ab^	3.1^bc^	2.8^c^	<0.001
Aftertaste	5.4	5.1	5.2	5.2	0.486

Recent studies have suggested that oral perception of dietary lipids constitutes a discrete basic taste modality, the oleogustus (Running et al., 2015; Yasumatsu et al., 2019). These studies indicate that the taste response of long chain fatty acids does not fully align with the expected sensation of ‘fattiness’ and shows some overlap with umami, which may help explain the pronounced meaty aroma and flavour observed in the crude sample.

The MF process mainly reduced meaty, roasted and rancid tastes and flavours, whereas vegetable taste and smell were more pronounced in the MF-permeate (Table 3). However, only minor additional effects on sensory attribute intensity were observed after NF, where only fatness was significantly reduced in the NF-dia product compared with MF. This contrasted with previous research, where NF significantly reduced the majority of sensory attributes found in fish protein hydrolysates (Steinsholm et al., 2021). Another interesting observation was the slight increase in bitter taste, across the filtration steps (significant between MF and NF-dia). This suggests that the small bitter peptides and hydrophobic amino acids were not removed in the NF steps. The NF step also reduced the ash content of the hydrolysate (Table 1), and, in particular, the removal of NaCl may contribute to this observation, as salt is known to mask or suppress bitterness by modulating taste receptor responses (Kumar & Behrens, 2024). This is consistent with previous findings showing that NaCl suppresses bitterness in protein hydrolysates by reducing peptide surface hydrophobicity and associated hydrophobic interactions (Xu et al., 2019). Still, it should be noted that part of the observed differences may also be influenced by the experimental design, as sensory assessment was performed at equal dry matter concentrations, while protein concentration increased after NF. This may have contributed to an increased perception of protein-related tastes and flavours, such as bitterness and umami.

### 3.3 Effect of membrane filtration on metabolite composition by ^1^H NMR

The samples were assessed by ^1^H NMR to reveal changes in their spectral profile that could be associated with the membrane filtration steps. The NMR spectra of all hydrolysates were comprised of broad signals from proteins and peptides, and sharp peaks assigned to free amino acids and abundant metabolites. The spectra revealed a complex peptide matrix with extensive signal overlap.

**Fig. 1:**
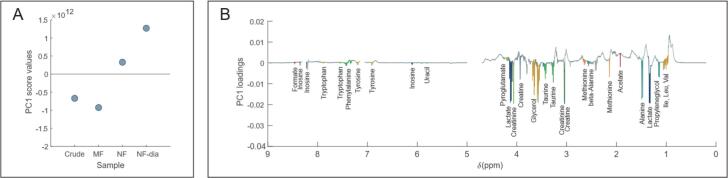
Principal Component Analysis (PCA) of NMR Spectra. **(A)** PCA scores plot (PC1) illustrating the effect of the filtration steps on sample composition. **(B)** Corresponding PC1 loading plot, highlighting spectral regions driving the separation. Positive loadings reflect broad signals associated with proteins and peptides enriched in the nanofiltration (NF) retentates, while negative loadings correspond to sharp signals from small metabolites and free amino acids, which were depleted during nanofiltration.

An unsupervised principal component analysis (PCA) of the spectra revealed the dominating chemical difference between the samples (Fig. 1). The first principal component (PC1) accounted for 91.2% of the total variance, indicating that the majority of the spectral variability across the samples was captured along this single dominant dimension. As expected, broad signals corresponding to proteins and peptides were enhanced in the NF retentates, as indicated by positive peaks in the PC1 loading vector (Fig. 1 Panel B). This is consistent with the chemical analyses (Table 1), which show both an increase in protein concentration and a shift toward higher molecular weight fractions in the size exclusion chromatography. In contrast, sharp signals associated with small metabolites and free amino acids were reduced, as indicated by negative peaks in the PC1 loadings (Fig. 1, Panel B), confirming that the applied filtration effectively removed low-molecular-weight compounds from the samples. The second principal component (PC2) accounted for 8.3% of the variance and primarily reflected concentration differences between samples along with a shift in the lactate peak position.

**Fig. 2:**
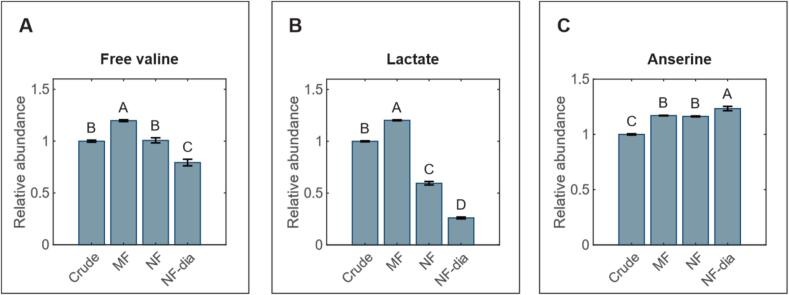
Relative abundance of (A) free valine, (B) lactate, and (C) anserine in the samples: crude hydrolysate (Crude), microfiltered (MF), and nanofiltered fractions (NF and NF-dia). Values are normalized to the abundance in the crude hydrolysate. Different letters indicate significant differences based on one-way ANOVA and Tukey's post hoc test (*p* < 0.05).

The simultaneous removal of a wide range of compounds introduces covariation in the spectra, complicating the attribution of sensory changes to individual metabolites. Notably, the concentrations of free amino acids typically associated with pronounced bitterness were reduced. For example, the relative abundance of free valine decreased following NF, as shown in Fig. 2, panel A. However, sensory evaluation paradoxically revealed an increase in perceived bitterness (Table 3), as discussed above. Although the concentrations of these amino acids were slightly reduced, they remained present at appreciable levels. A plausible explanation is that compounds with bitterness-masking properties were also eliminated during filtration. These include NMR-detectable metabolites such as organic acids (e.g., lactate (Fig. 2, panel B), acetate, and formate, NaCl, as discussed above, and ^1^H NMR-invisible salts. The dipeptide anserine was up concentrated after NF (Fig. 2, panel C), indicating that it was retained by the NF membrane. Anserine has previously been associated with several sensory attributes, including fullness, sweetness, saltiness, umami, sourness, and fatness (Steinsholm et al., 2020). However, in the present study, only sour taste increased significantly following NF (Table 3). This suggests that the contribution of individual metabolites to sensory perception is highly context-dependent and that the observed sensory changes are governed by interactions within the hydrolysate matrix rather than by single compounds alone.

### 3.4 Effect of membrane filtration on volatile compounds

HS-GC/MS was used to assess the effect of membrane filtration on lipid-oxidation products and other odour active volatile compounds originating from the crude hydrolysate. The hydrolysates were dominated by aldehydes, ketones and esters (Supplementary Table 2), characterized by lipid and protein oxidation products. Crude hydrolysate contained the highest levels of oxidation products, which were reduced by 34%, (MF), 42% (NF) and 48% (NF-dia). Hexanal had the highest abundance in the crude fraction, however this was subsequently reduced by 94–96% after filtration. Hexanal is widely recognized as a marker of lipid oxidation (Li et al., 2024), and associated with undesirable sensory attributes, notably grassy and vegetable-like notes (Leksrisompong et al., 2010). Further, 13 volatile compounds, mostly secondary lipid oxidation products were removed entirely after filtration.

**Fig. 3:**
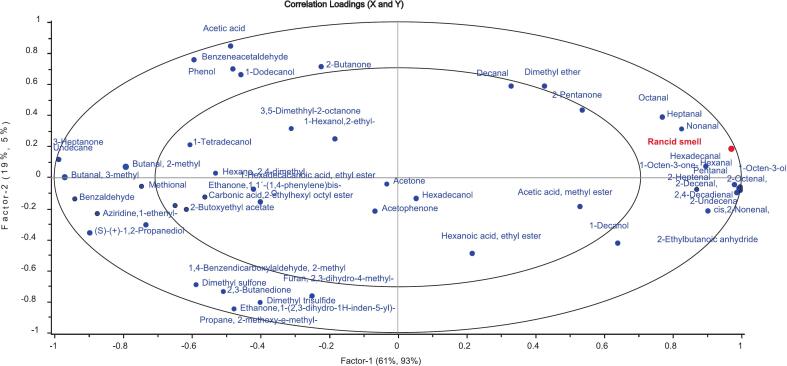
PLS correlation loading plot showing relationships between volatile compounds and rancid smell in poultry protein hydrolysates. Compounds located in the same direction are positively correlated. Significant correlations were observed with rancid attributes (*r* = 0.60 and 0.58, *n* = 4, *p* < 0.025).

PLS regression models based on the volatile odour compounds were investigated for the significant (*p* < 0.001) sensory attributes meaty and rancid smell and flavour. The correlation model outcome showed that the same volatile compounds could be associated with both rancid smell and taste, as shown for the rancid smell in Fig. 3, (the correlation was significant, respectively *r* = 0.60 (*n* = 4, *p* < 0.025) for rancid smell and *r* = 0.58 (n = 4, p < 0.025) for rancid taste). All the lipid oxidation products are associated with the rancid smell (far right, Fig. 3), which can be explained by the fact that most of these compounds have rancid notes related to grass, hay, wax, fat, paint and soap (notes used in our sensory definition of rancid). Considering also the odour thresholds of these lipid oxidation products in water, the oxidation products with the highest odour activity contributing to rancid smell are, in decreasing order, decanal, nonanal, tr,cis, 2,4-decadienal, cis-2-nonenal and hexanal. The correlation outcome for the meaty smell and taste showed similar variable loading distribution as for the rancid attributes with a significant correlation, r = 0.58 (n = 4, p < 0.025) to 0.53 (n = 4, p < 0.025). It has earlier been reported that the smell of tr,2-decenal and tr,tr-2,4-decadienal may also contribute to meaty character (Cui et al., 2023; Mottram, 1998).

Protein oxidation should also be considered when evaluating the effects of membrane filtration, as it may contribute to the formation of key flavour compounds. Several Strecker degradation products were identified, including methional, 2-methylbutanal and 3-methylbutanal, originating from methionine (Xiong & Guo, 2021), isoleucine and leucine (Huang et al., 2017), respectively. 2-Methylbutanal and 3-methylbutanal have been described as contributing malty, chocolate-like and meat-related notes (Arsa & Theerakulkait, 2018; Hellwig, 2020), yet showed poor correlation with the meaty sensory attributes in the present study. Interestingly, the crude sample exhibited the lowest levels of protein oxidation markers, whereas filtered samples showed 25–45% higher levels (Supplementary Table 2). This increase may reflect either the higher relative protein content or enhanced formation of oxidation products during processing and storage. However, the lack of a clear relationship between protein concentration and oxidation marker levels suggests that formation during processing is the more likely explanation. However, no clear relationship between protein concentration and the abundance of these compounds was observed, suggesting that their formation is influenced by multiple factors beyond simple concentration effects. Although these compounds are typically small enough to permeate NF membranes, their retention in the filtered fractions may indicate interactions with the matrix, including membrane fouling effects or post-filtration formation.

Still, the role of individual volatile compounds in explaining specific sensory changes remains limited. For example, methional increased after filtration and has been associated with potato-like and vegetable-like notes (Leksrisompong et al., 2010; The Good Scents Company Information System, 2026) but did not correlate with the meaty attributes in this study. This suggests that the increased perception of vegetable-like flavour after filtration is not driven by a single compound, but rather by changes in the overall balance of flavour compounds. This is consistent with previous studies on chicken broth, which demonstrate that flavour perception results from the combined contribution of multiple compound classes, including amino acids, small metabolites, and lipid oxidation products (Jia et al., 2023).

Taken together, the results indicate that flavour perception is governed by interactions within the matrix, where removal of dominant flavour contributors (e.g., lipid oxidation products) may enhance the perception of more subtle notes, rather than directly reflecting the concentration of individual volatiles. The apparent contradiction between reduced free amino acids observed by ^1^H NMR and the increased bitterness perceived after NF highlights this complexity. Bitterness is therefore not solely determined by the concentration of bitter compounds, but also by the presence of taste-modulating components. The removal of sodium chloride and organic acids during NF may reduce masking effects and thereby enhance bitterness, even at lower levels of free amino acids. In addition, the increased protein concentration after NF may have contributed to higher levels of peptides per serving. Together, these results indicate that sensory changes following NF primarily reflect shifts in matrix composition and taste interactions rather than direct changes in individual bitter metabolites.

## 4 Conclusions

The use of membrane filtration significantly reduced the sensory intensity of poultry protein hydrolysates, with most taste and flavour attributes decreasing after MF. In contrast, NF and diafiltration primarily affected chemical composition, reducing levels of small metabolites without markedly improving overall sensory attributes. Interestingly, NF led to a slight increase in perceived bitterness, likely reflecting a combination of increased protein concentration and removal of bitter-masking compounds such as salts. Analysis of volatile compounds confirmed that lipid oxidation products, including hexanal, were substantially reduced after MF, supporting its role in mitigating off-flavours. Overall, these findings provide a better understanding of how membrane filtration shapes both composition and sensory perception in protein hydrolysates and may support the development of more tailored processing strategies for improving their applicability in food systems.

## Declaration of generative AI and AI-assisted technologies in the manuscript preparation process

During the preparation of this work, the authors used Microsoft Copilot for language refinement and Consensus for reference searching. After using these tools, the authors reviewed and edited the content as needed and takes full responsibility for the content of the published article.

## CRediT authorship contribution statement

**Tone Aspevik:** Writing – review & editing, Writing – original draft, Methodology, Investigation, Formal analysis, Data curation, Conceptualization. **Elin Katinka Dankel:** Writing – review & editing, Writing – original draft, Software, Methodology, Investigation, Formal analysis, Data curation. **Shaun Leivers:** Writing – review & editing, Writing – original draft, Software, Methodology, Formal analysis, Data curation. **Nils Kristian Afseth:** Writing – review & editing, Validation, Supervision, Methodology. **Åge Oterhals:** Writing – review & editing, Validation, Supervision, Investigation, Conceptualization. **John-Erik Haugen:** Writing – review & editing, Validation, Supervision, Methodology. **Marianne Skov:** Writing – review & editing, Supervision, Project administration, Funding acquisition. **Mari Øvrum Gaarder:** Writing – review & editing, Validation, Supervision, Project administration, Methodology, Funding acquisition, Conceptualization.

## Declaration of competing interest

The authors declare that they have no known competing financial interests or personal relationships that could have appeared to influence the work reported in this paper.

## Data Availability

Data will be made available on request.
