# Supplementary material for: Upcycling of poultry protein hydrolysates using membrane filtration technology – Effects on sensory properties and chemical composition
Source: Food Chem X. 2026 Jul 8;38:104181. doi: 10.1016/j.fochx.2026.104181 (PMC13425802; doi:10.1016/j.fochx.2026.104181)
Supplement: Supplementary file 1 — Supplementary material 1 [file mmc1.docx]

**Supplementary Table 1**: Sensory attributes used in the sensory evaluation of the protein hydrolysates.

| Attribute | Description |
| --- | --- |
| Total intensity of smell | The strength of all odours and smells in the sample |
| Vegetable smell | Related to the smell and odour of grain, potato, bread, nuts, potato water |
| Meaty smell | Related to smells and oduors reminding of animal flesh of pork and chicken |
| Process smell | Related to smells and odours like diesel, mechanic motor, engine oil, rubber |
| Roasted smell | Related to smells and odours from fried meat |
| Rancid smell | The intensity of rancid smells and odours like grass, hay, stearine, paint, oxidised fat |
| Total intensity of taste and flavour | The strength of all flavours in the sample |
| Sweet taste | Related to the basic taste sweet (sucrose) |
| Salty taste | Related to the basic taste salt (sodium chloride) |
| Acidic taste | Related to the basic taste acidic (citric acid) |
| Bitter taste | Related to the basic taste bitter (caffeine) |
| Umami taste | Related to the basic taste umami (monosodium glutamate) |
| Vegetable flavour | Related to the flavours of grain, potato, bread, nuts, potato water |
| Meaty flavour | Related to flavours reminding of animal flesh of pork and chicken |
| Process flavour | Related to flavours like diesel, mechanic motor, engine oil, rubber |
| Roasted flavour | Related to flavours of fried meat |
| Rancid flavour | The intensity of rancid flavours like grass, hay, stearine, paint, oxidised fat |
| Fatness | Surface textural attribute relating to perception of the quantity of fat in the product |
| After taste | Taste which occurs 15 seconds after elimination of the product |
