# Supplementary material for: Upcycling of poultry protein hydrolysates using membrane filtration technology – Effects on sensory properties and chemical composition
Source: Food Chem X. 2026 Jul 8;38:104181. doi: 10.1016/j.fochx.2026.104181 (PMC13425802; doi:10.1016/j.fochx.2026.104181)
Supplement: Supplementary file 2 — Supplementary material 2 [file mmc2.docx]

**Supplementary Table 2**: Mean concentration of volatile organic compounds in ng/ml of two replicates. Precision within ≤ 15 %.

| Compound Name | Crude | MF | NF | NF-dia |
| --- | --- | --- | --- | --- |
| Propane, 2-methoxy-2-methyl- | nd | nd | nd | 0.95 |
| Hexane, 2,4-dimethyl- | nd | 0.67 | nd | 0.47 |
| Acetone | 3.34 | 1.93 | 4.18 | 3.35 |
| 2-Butanone | 17.79 | 30.53 | 17.87 | 17.03 |
| Butanal, 2-methyl- | 4.49 | 7.05 | 5.51 | 6.72 |
| Butanal, 3-methyl- | 11.40 | 15.94 | 14.15 | 16.56 |
| 2,3-Butanedione | nd | 1.38 | nd | 1.19 |
| Dimethyl ether | 13.63 | 15.87 | 7.33 | 7.57 |
| 2-Pentanone | 2.33 | 0.92 | 2.24 | nd |
| 3-Heptanone, 5-ethyl-4-methyl- | nd | 2.57 | 2.68 | 2.49 |
| Pentanal | 4.07 | nd | nd | nd |
| Acetic acid ethenyl ester | nd | 4.50 | 3.37 | nd |
| 2,3-Butanedione | nd | nd | nd | 3.45 |
| Aziridine, 1-ethenyl- | nd | 0.46 | 1.13 | 1.33 |
| Dimethyl sulfone | nd | nd | nd | 1.10 |
| Hexanal | 24.28 | 1.44 | 1.47 | 1.05 |
| Undecane | 2.23 | 4.07 | 3.86 | 4.02 |
| Heptanal | 1.70 | 0.85 | 1.10 | 0.69 |
| Furan, 2,3-dihydro-4-methyl- | 1.03 | 0.82 | 1.16 | 1.33 |
| Hexanoic acid, ethyl ester | 0.58 | 0.57 | 0.57 | 0.58 |
| Octanal | 2.81 | 1.65 | 2.01 | 1.18 |
| 1-Octen-3-one | 1.47 | nd | nd | nd |
| Acetic acid, methyl ester | 4.53 | nd | 4.20 | 2.24 |
| tr, 2-Heptenal | 0.84 | nd | nd | nd |
| 2-Ethylbutanoic anhydride | 1.55 | nd | nd | nd |
| Dimethyl trisulfide | nd | nd | nd | 0.94 |
| Nonanal | 8.11 | 0.61 | 0.60 | 0.26 |
| tr, 2-Octenal | 1.46 | nd | nd | nd |
| 1-Octen-3-ol | 4.03 | nd | nd | nd |
| 1-Hexanol, 4-methyl- | 0.43 | nd | 0.13 | nd |
| 2-Butoxyethyl acetate | 0.57 | nd | nd | nd |
| Methional | 4.96 | 5.51 | 7.64 | 6.43 |
| 3-Furaldehyde | 0.91 | nd | nd | nd |
| 3,5-Dimethyl-2-octanone | nd | nd | 1.21 | nd |
| 1-Hexanol, 2-ethyl- | 1.64 | 1.47 | 2.66 | 1.63 |
| Decanal | 4.28 | 3.06 | 4.85 | 1.75 |
| Benzaldehyde | 12.16 | 15.52 | 15.60 | 17.81 |
| cis, 2-Nonenal | 0.32 | nd | nd | nd |
| 1-Octanol | 0.59 | 0.30 | 0.34 | 0.24 |
| tr, 2-Decenal | 0.47 | nd | nd | nd |
| Benzeneacetaldehyde | 9.20 | 12.06 | 11.07 | 9.71 |
| Acetophenone | 3.20 | 2.01 | 3.81 | 3.62 |
| 2-Undecenal | 0.49 | nd | nd | nd |
| (S)-(+)-1,2-Propanediol | nd | 0.80 | 0.87 | 1.40 |
| tr,tr, 2,4-Decadienal | 2.87 | nd | nd | nd |
| 1,4-Benzenedicarboxaldehyde, 2-methyl- | 0.93 | 0.91 | 1.04 | 1.14 |
| 2,2,4-Trimethyl-1,3-pentanediol diisobutyrate | 4.69 | 4.47 | 6.12 | 6.95 |
| 1-Dodecanol | nd | 0.34 | 0.69 | 0.24 |
| 1-Decanol | 0.87 | 0.33 | nd | 0.50 |
| Ethanone, 1-(2,3-dihydro-1H-inden-5-yl)- | nd | nd | 1.79 | 6.41 |
| Phenol | nd | 4.28 | 7.40 | nd |
| Hexadecanal | 4.29 | nd | nd | nd |
| 1-Tetradecanol | 0.54 | 0.69 | 2.21 | 1.16 |
| Hexadecanoic acid, ethyl ester | 1.90 | nd | 3.93 | 2.24 |
| Carbonic acid, 2-ethylhexyl octyl ester | 0.54 | 0.35 | 1.26 | 1.02 |
| Ethanone, 1,1'-(1,4-phenylene)bis- | 0.67 | nd | 3.94 | 2.15 |
| 1-Hexadecanol | 1.21 | 0.89 | 2.70 | 1.77 |

nd = not detected
